# Messrs. P. Blakiston & Co.

**Published:** 1893-10

**Authors:** 


### BOOK NOTICES.

Messrs. P. Blakiston, Son & Co., Philadelphia, desire to announce A NEW ILLUSTRATED DICTIONARY OF MEDICINE, BIOLOGY, AND COLLATERAL SCIENCES, by Dr. George M. Gould, already well known as the editor of two small medical dictionaries, has now about ready an unabridged, exhaustive work of the same class, upon which he and a corps of able assistants have been uninterruptedly engaged for several years.

It contains a large number of fine illustrations that have been included, many of which—as, for instance, the series of over fifty of the bacteria—have been drawn and engraved especially for the work. It contains a far larger number of words than any other one-volume medical lexicon. It is a new book, not a revision of the older volume. The pronunciation, etymology, definition, illustration, and logical groupings of each word are given.

